# Soft Coral-Derived Dihydrosinularin Exhibits Antiproliferative Effects Associated with Apoptosis and DNA Damage in Oral Cancer Cells

**DOI:** 10.3390/ph14100994

**Published:** 2021-09-29

**Authors:** Kun-Han Yang, Yu-Sheng Lin, Sheng-Chieh Wang, Min-Yu Lee, Jen-Yang Tang, Fang-Rong Chang, Ya-Ting Chuang, Jyh-Horng Sheu, Hsueh-Wei Chang

**Affiliations:** 1 Graduate Institute of Natural Products, Kaohsiung Medical University, Kaohsiung 80708, Taiwan; R100024@kmu.edu.tw (K.-H.Y.); aaronfrc@kmu.edu.tw (F.-R.C.); 2 Department of Biomedical Science and Environmental Biology, PhD Program in Life Science, College of Life Science, Kaohsiung Medical University, Kaohsiung 80708, Taiwan; mark10395@gmail.com (Y.-S.L.); u107851101@gap.kmu.edu.tw (S.-C.W.); u107023047@kmu.edu.tw (M.-Y.L.); u107023007@gap.kmu.edu.tw (Y.-T.C.); 3 School of Post-Baccalaureate Medicine, Kaohsiung Medical University, Kaohsiung 80708, Taiwan; reyata@kmu.edu.tw; 4 Department of Radiation Oncology, Kaohsiung Medical University Hospital, Kaohsiung 80708, Taiwan; 5 Department of Marine Biotechnology and Resources, National Sun Yat-sen University, Kaohsiung 80424, Taiwan; 6 Doctoral Degree Program in Marine Biotechnology, National Sun Yat-sen University, Kaohsiung 80424, Taiwan; 7 Department of Medical Research, China Medical University Hospital, China Medical University, Taichung 40402, Taiwan; 8 Frontier Center for Ocean Science and Technology, National Sun Yat-sen University, Kaohsiung 80424, Taiwan; 9 Institute of Medical Science and Technology, National Sun Yat-sen University, Kaohsiung 80424, Taiwan; 10 Center for Cancer Research, Kaohsiung Medical University, Kaohsiung 80708, Taiwan; 11 Department of Medical Research, Kaohsiung Medical University Hospital, Kaohsiung 80708, Taiwan

**Keywords:** soft coral, Dihydrosinularin (DHS), oral cancer, apoptosis, DNA damage, reactive oxygen species (ROS), MitoMP, MitoSOX

## Abstract

Dihydrosinularin (DHS) is an analog of soft coral-derived sinularin; however, the anticancer effects and mechanisms of DHS have seldom been reported. This investigation examined the antiproliferation ability and mechanisms of DHS on oral cancer cells. In a cell viability assay, DHS showed growth inhibition against several types of oral cancer cell lines (Ca9-22, SCC-9, OECM-1, CAL 27, OC-2, and HSC-3) with no cytotoxic side effects on non-malignant oral cells (HGF-1). Ca9-22 and SCC-9 cell lines showing high susceptibility to DHS were selected to explore the antiproliferation mechanisms of DHS. DHS also causes apoptosis as detected by annexin V, pancaspase, and caspase 3 activation. DHS induces oxidative stress, leading to the generation of reactive oxygen species (ROS)/mitochondrial superoxide (MitoSOX) and mitochondrial membrane potential (MitoMP) depletion. DHS also induced DNA damage by probing γH2AX phosphorylation. Pretreatment with the ROS scavenger *N*-acetylcysteine (NAC) can partly counter these DHS-induced changes. We report that the marine natural product DHS can inhibit the cell growth of oral cancer cells. Exploring the mechanisms of this cancer cell growth inhibition, we demonstrate the prominent role DHS plays in oxidative stress.

## 1. Introduction

One of the high-incidence malignancies worldwide, oral cancer, presents a significant public health problem [1]. According to Cancer Statistics 2021, the incidence and death rates for oral cancer are increasing for both men and women [2]. The five-year survival rate is higher in young individuals than in old individuals [3]. Besides surgery, radiotherapy and chemotherapy are common ways to treat oral cancer, but they are frequently associated with severe side effects [4]. Developing more anticancer drugs with fewer side effects or preferential antiproliferation effects is necessary for improved oral cancer therapy.

Natural products may potentially prevent or reduce the side effects of oral cancer chemotherapy and radiotherapy [5]. Marine natural products exhibit diverse functions to avoid damage from bacterial, fungal, protozoal, tuberculosis, and viral infections [6,7,8,9,10]. Many extracts and compounds derived from marine natural products such as sponges and soft corals also show anticancer effects [8,9,11,12,13,14,15]. These extracts warrant further development as anticancer drugs for oral cancer therapy.

Soft corals are abundant resources for anticancer drugs [7,9,16,17]. Soft corals such as *Sinularia flexibilis* (*S. flexibilis*) are widely distributed in the western Pacific and eastern Indian Oceans. *S. manaarensis* is a kind of Formosan soft coral [18]. *S. flexibilis* and *S. sandensis* can be cultivated in Taiwan [19]. All these soft corals are commonly used for bioactive compound isolation [18,19].

Although sinularin and dihydrosinularin (DHS) were identified at the same time [20], most anticancer studies of *Sinularia* focus on sinularin [21,22,23,24,25]. The structural difference between sinularin and DHS is that sinularin provides an additional conjugated double bond compared to DHS [26]. Moreover, sinularin shows higher antioxidant abilities than DHS. However, the anticancer effects of DHS have rarely been investigated to date. The cytotoxic effects of DHS have been reported in several cancer cell lines such as lung cancer, colon cancer, and leukemia [26,27]. Still, these studies only provided IC_50_ values without a detailed investigation of the mechanism of its anticancer effect [26]. Additionally, the anticancer effect of this compound on oral cancer cells remains unclear. Several kinds of oral cancer cell lines have been established, which were derived from the gingival, tongue, and buccal locations. The discovery of anticancer drugs for the treatment of different types of oral cancer cells warrants investigation.

In the present study, we aimed to evaluate the antiproliferation potential of DHS on oral cancer cells using several types of oral cancer cell lines and a non-malignant oral cell line. The mechanisms of antiproliferation of DHS against oral cancer cells were also investigated.

## 2. Results

### 2.1. DHS Kills Oral Cancer Cells Alleviated by NAC

**Figure 1:**
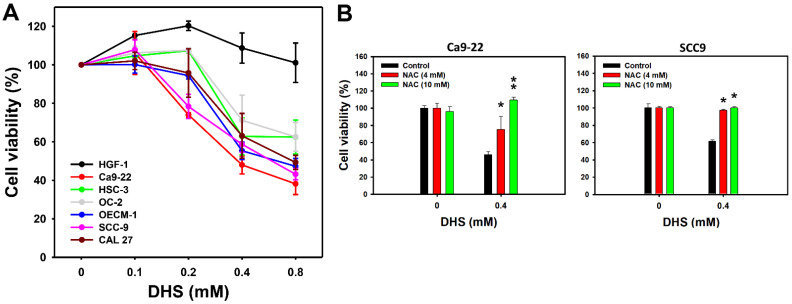
DHS kills oral cancer cells. (**A**) MTS assay (48 h) for DHS-treated oral cancer and non-malignant oral cells. Six oral cancer cell lines (Ca9-22, SCC-9, OECM-1, CAL 27, OC-2, and HSC-3) and one non-malignant oral cell line (HGF-1) were chosen. Cells were treated with DHS (0 (vehicle containing 0.08% DMSO), 0.1, 0.2, 0.4 and 0.8 mM) for 48 h. (**B**) Recovery of cell viability of DHS-treated Ca9-22 and SCC-9 cells by NAC. Cells were pretreated with NAC (10 mM for 1 h) or not. They were then treated with 0.4 mM DHS or with the vehicle for 0 and 48 h. Data = means ± SDs (*n* = 3 independent experiments). * and ** indicate significant differences between drug treatments (NAC/DHS) and DHS (*p* < 0.05 and 0.01, respectively).

A 48 h MTS assay determined the cell viabilities of oral cancer and non-malignant oral cells following DHS treatment. The cell viabilities of a panel of oral cancer cell lines, derived from gingival (Ca9-22 and OCEM-1), tongue (SCC-9, CAL 27, and HSC-3), and buccal (OC-2) locations, were decreased by DHS treatment for 48 h (Figure 1A). In comparison, the cell viability of non-malignant oral cells (HGF-1) remained similar to the control and exhibited no cytotoxicity. These results indicate that DHS exhibits antiproliferative potential for oral cancer cells. According to cell viability, two oral cancer cell lines, Ca9-22 and SCC-9, were susceptible to DHS. These were therefore selected for the subsequent experiments exploring the antiproliferation mechanisms of DHS.

To examine the oxidative stress function of DHS-induced antiproliferation, pretreatment with the reactive oxygen species (ROS) scavenger *N*-acetylcysteine (NAC) [28] was applied before DHS posttreatment. NAC pretreatment alleviated the DHS-induced antiproliferative effect on Ca9-22 and SCC-9 cells (Figure 1B), suggesting that DHS induces antiproliferation of oral cancer cells in a ROS-dependent manner.

### 2.2. DHS Accumulates SubG1 Phase of Oral Cancer Cells Alleviated by NAC

**Figure 2:**
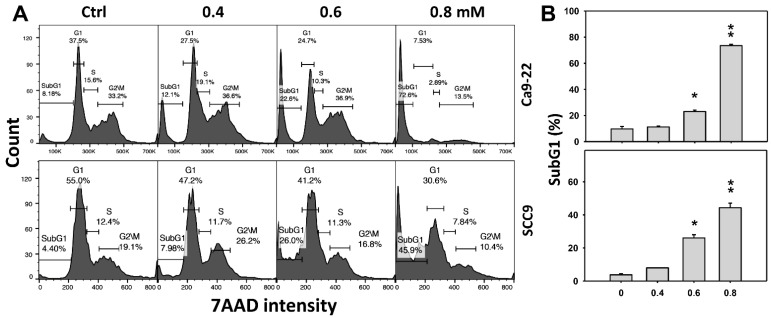

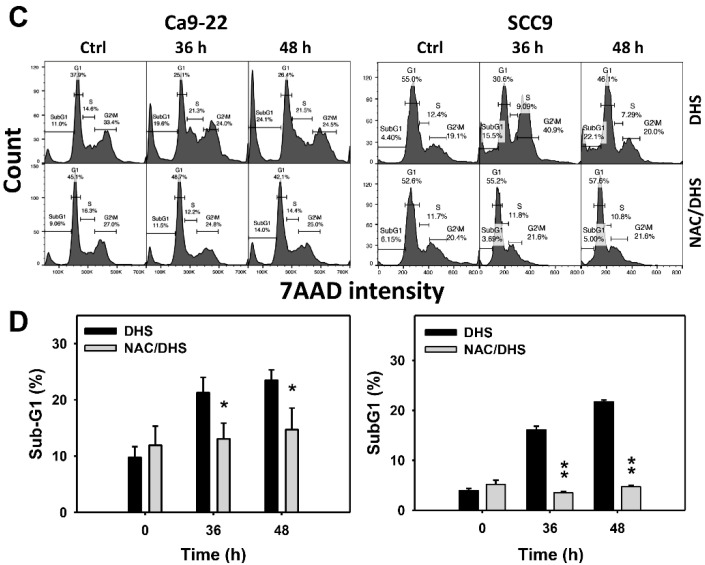
DHS effects on subG1 accumulation of oral cancer cells. (**A**,**B**) Representative cell cycle patterns of DHS-treated oral cancer cells. Cells (Ca9-22 and SCC-9) were treated with 0.4, 0.6 and 0.8 mM DHS or vehicle (containing 0.08% DMSO) for 48 h. (**C**,**D**) Alleviation of subG1 accumulation of DHS-treated oral cancer cells by NAC. Cells were pretreated with NAC (4 mM, 1 h) or not, and then they were treated with 0.6 mM DHS or vehicle for 0, 36 and 48 h. Data = means ± SDs (*n* = 3 independent experiments). * and ** indicate significant differences between (**A**,**B**) DHS and control and (**C**,**D**) DHS and NAC/DHS (*p* < 0.05 and 0.01, respectively). A positive control for subG1 accumulation of oral cancer cells is provided in Supplementary Figure S1.

Cell cycle change is a common biomarker of cellular drug response. The concentration effect of DHS on cell cycle patterns in oral cancer cells was demonstrated (Figure 2A). subG1 events of oral cancer cells (Ca9-22 and SCC-9) were increased by DHS treatment (Figure 2B). Moreover, NAC impact on time course revealed cell cycle changes in oral cancer cells following DHS treatment (Figure 2C). The subG1 events of Ca9-22 and SCC-9 cells were increased from 0 h to 48 h by DHS treatment (Figure 2D). After NAC pretreatment, DHS-induced subG1 accumulations were slightly suppressed in Ca9-22 cells but substantially suppressed in SCC-9 cells, suggesting that DHS differentially induced subG1 accumulation of oral cancer cells in a ROS-dependent manner.

### 2.3. DHS Induces Annexin V-Detected Apoptosis of Oral Cancer Cells Alleviated by NAC

**Figure 3:**
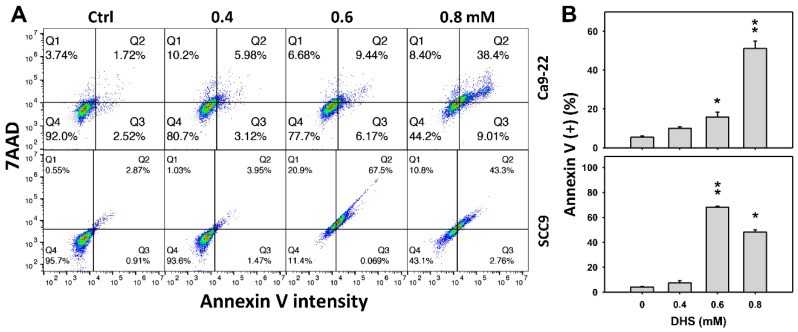

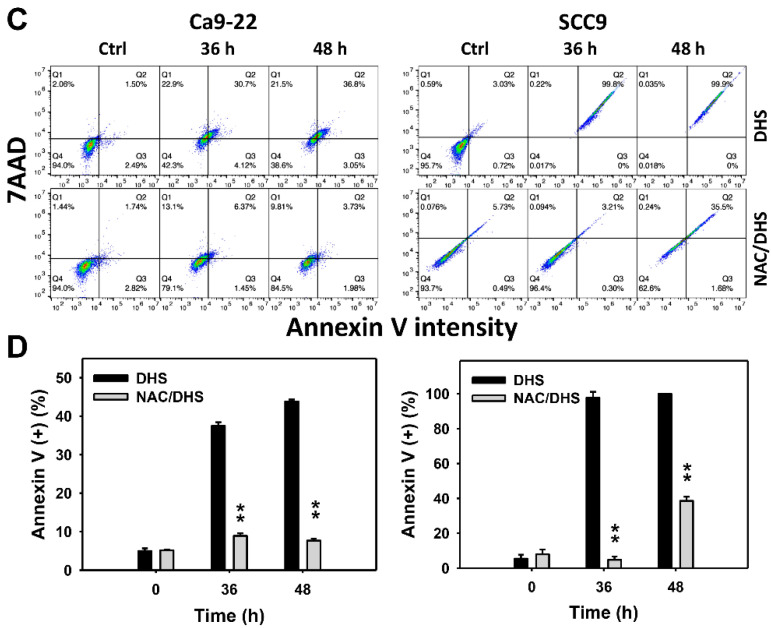
DHS effects on annexin V/7AAD-detected apoptosis of oral cancer cells. (**A**,**B**) Representative annexin V/7AAD patterns of DHS-treated oral cancer cells. Cells (Ca9-22 and SCC-9) were treated with 0.4, 0.6 and 0.8 mM DHS or vehicle (containing 0.08% DMSO) for 48 h. (**C**,**D**) Alleviation of the annexin V/7AAD changes of DHS-treated oral cancer cells by NAC. Cells were pretreated with NAC (4 mM, 1 h) or not, and then they were treated with 0.6 mM DHS or vehicle for 0, 36 and 48 h. Data = means ± SDs (*n* = 3 independent experiments). * and ** indicate significant differences between (**A**,**B**) DHS and control and (**C**,**D**) DHS and NAC/DHS (*p* < 0.05 and 0.01, respectively). A positive control for annexin V/7AAD-detected apoptosis of oral cancer cells is provided in Supplementary Figure S1.

The apoptotic effect of DHS was primarily evaluated by annexin V/7-aminoactinmycin D (7AAD) analysis. The concentration effect of DHS on the annexin V/7AAD pattern in oral cancer cells was demonstrated (Figure 3A). The annexin V (+)/7AAD (+/−) events of oral cancer cells (Ca9-22 and SCC-9) were increased by DHS treatment (Figure 3B). Moreover, a NAC impact on the time course of annexin V changes in oral cancer cells following DHS treatment was demonstrated (Figure 3C). The annexin V (+)/7AAD (+/−) events of Ca9-22 and SCC-9 cells were increased from 0 h to 48 h by DHS treatment (Figure 2D). After NAC pretreatment, DHS-induced apoptosis was suppressed, suggesting that DHS induces apoptosis in oral cancer cells in a ROS-dependent manner.

### 2.4. Alleviation by NAC of Apoptosis of Oral Cancer Cells as Detected According to DHS-Induced Caspase Activation

**Figure 4:**
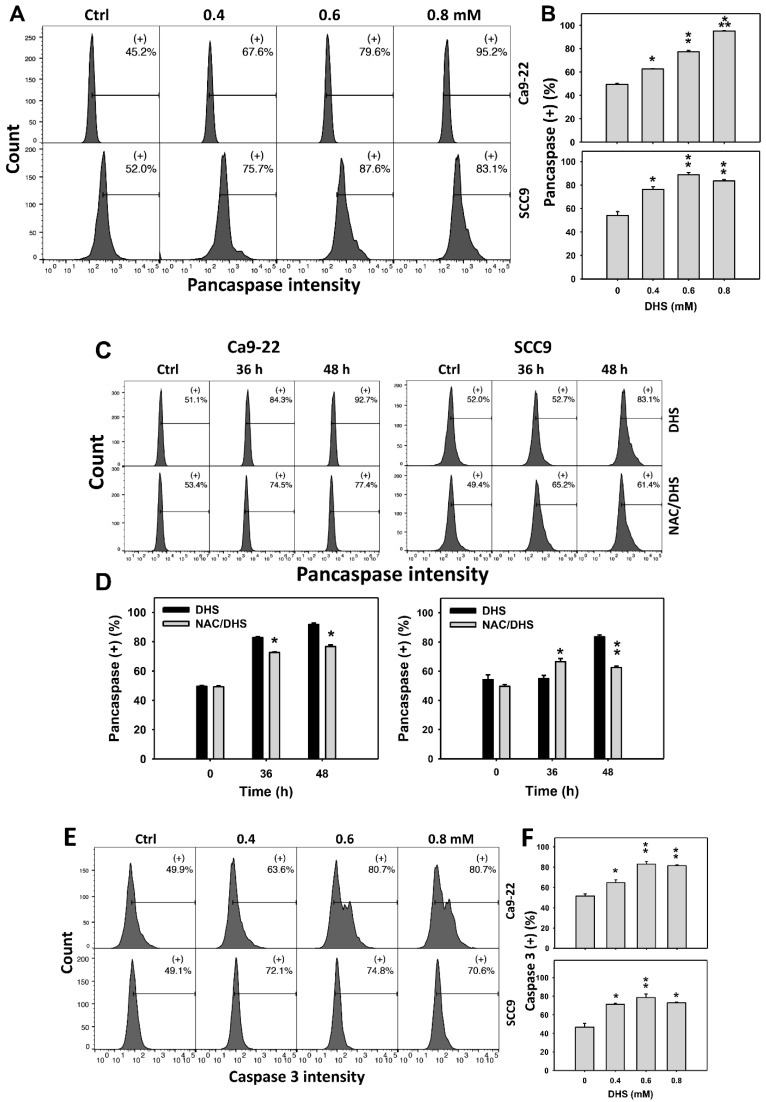
DHS effects on caspase-based apoptosis of oral cancer cells. (**A**,**B**) Representative pancaspase patterns of DHS-treated oral cancer cells. Cells (Ca9-22 and SCC-9) were treated with vehicle (containing 0.08% DMSO), 0.4, 0.6 and 0.8 mM DHS for 48 h. (+) indicates the high pancaspase activity populations. (**C**,**D**) Alleviation of the pancaspase changes of DHS-treated oral cancer cells by NAC. Cells were pretreated with NAC (4 mM, 1 h) or not, and then they were treated with the vehicle and 0.6 mM DHS for 0, 36 and 48 h. (**E**,**F**) Representative caspase 3 patterns of DHS-treated oral cancer cells. Data = means ± SDs (*n* = 3). *, ** and *** indicate significant differences between (**A**,**B**) DHS and control and (**C**,**D**) DHS and NAC/DHS (*p* < 0.05, 0.01, and 0.001, respectively). A positive control for pancaspase-detected apoptosis of oral cancer cells is provided in Supplementary Figure S1.

The apoptotic effect of DHS on oral cancer cells was evaluated by monitoring the pancaspase activity. The concentration effect of DHS on pancaspase patterns in oral cancer cells was demonstrated (Figure 4A). The pancaspase (+) events of oral cancer cells (Ca9-22 and SCC-9) were increased by DHS treatment (Figure 4B). Moreover, the NAC impact on time course pancaspase changes in oral cancer cells following DHS treatment were demonstrated (Figure 4C). The pancaspase (+) events of Ca9-22 and SCC-9 cells were increased from 0 h to 48 h by DHS treatment (Figure 4D). After NAC pretreatment, the DHS-induced pancaspase activations were slightly suppressed for Ca9-22 cells but slightly induced for SCC-9 cells after 36 h. After NAC pretreatment, the DHS-induced pancaspase activations were partly suppressed after 48 h. These results suggest that DHS induced pancaspase activation in oral cancer cells after 48 h of treatment in a ROS-dependent manner.

In addition, these pancaspase activations were further examined by specific caspases such as caspase 3 flow cytometry (Figure 4E). The caspase 3 (+) events of oral cancer cells (Ca9-22 and SCC-9) were increased by DHS treatment (Figure 4F). These results suggest that DHS triggered caspase 3 activation in the apoptosis of oral cancer cells.

### 2.5. DHS Induces Reactive Oxygen Species (ROS) Stress of Oral Cancer Cells Alleviated by NAC

**Figure 5:**
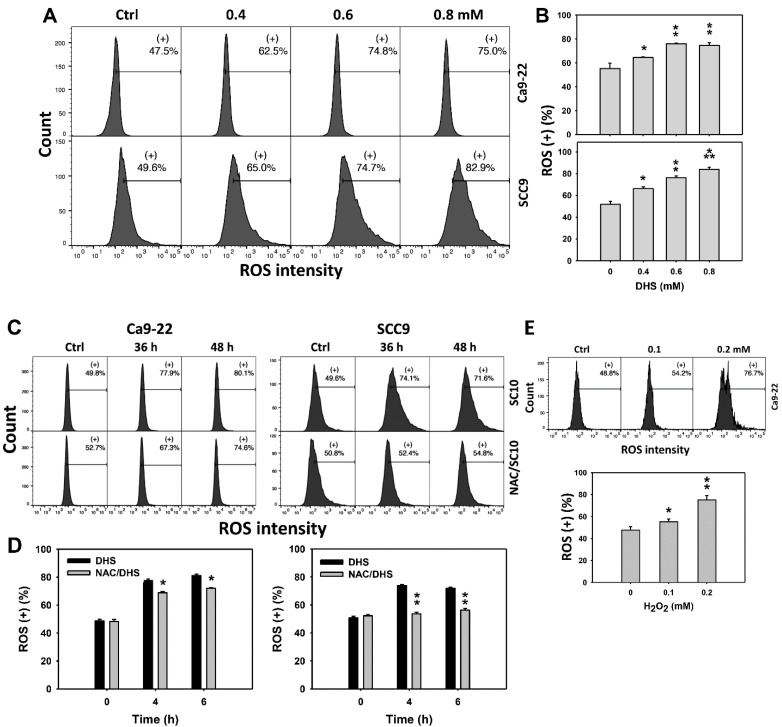
DHS effects on ROS contents of oral cancer cells. (**A**,**B**) Representative ROS patterns of DHS-treated oral cancer cells. Cells (Ca9-22 and SCC-9) were treated with vehicle (containing 0.08% DMSO), 0.4, 0.6 and 0.8 mM DHS for 48 h. (+) indicates the high ROS populations. (**C**,**D**) Alleviation of ROS changes in DHS-treated oral cancer cells by NAC. Cells were pretreated with NAC (4 mM, 1 h) or not, and then they were treated with 0.6 mM DHS or vehicle for 0, 36 and 48 h. (**E**) Positive control for ROS induction. Cells (Ca9-22) were treated with H_2_O_2_ (0, 0.1 and 0.2 mM) for 24 h. Data = means ± SDs (*n* = 3 independent experiments). *, ** and *** indicate significant differences between (**A**,**B**) DHS and control and (**C**,**D**) DHS and NAC/DHS (*p* < 0.05, 0.01 and 0.001, respectively).

Since ROS are commonly involved in triggering apoptosis [29], the ROS induction of DHS-treated oral cancer cells was evaluated. The concentration effect of DHS on ROS pattern in oral cancer cells was demonstrated (Figure 5A). The ROS (+) events of oral cancer cells (Ca9-22 and SCC-9) were increased by DHS treatment (Figure 5B). Moreover, the NAC impact of time course ROS changes in oral cancer cells following DHS treatment were demonstrated (Figure 5C). The ROS (+) events of Ca9-22 and SCC-9 cells were increased from 0 h to 48 h by DHS treatment (Figure 5D). For comparison, H_2_O_2_ showed ROS generation as a positive control (Figure 5E). After NAC pretreatment, DHS-induced ROS stresses were slightly suppressed in Ca9-22 cells but dramatically suppressed in SCC-9 cells, suggesting that NAC suppresses more DHS-induced ROS stress in oral cancer cells (SCC-9) than Ca9-22 cells.

### 2.6. DHS Induces MitoSOX Stress of Oral Cancer Cells Alleviated by NAC

**Figure 6:**
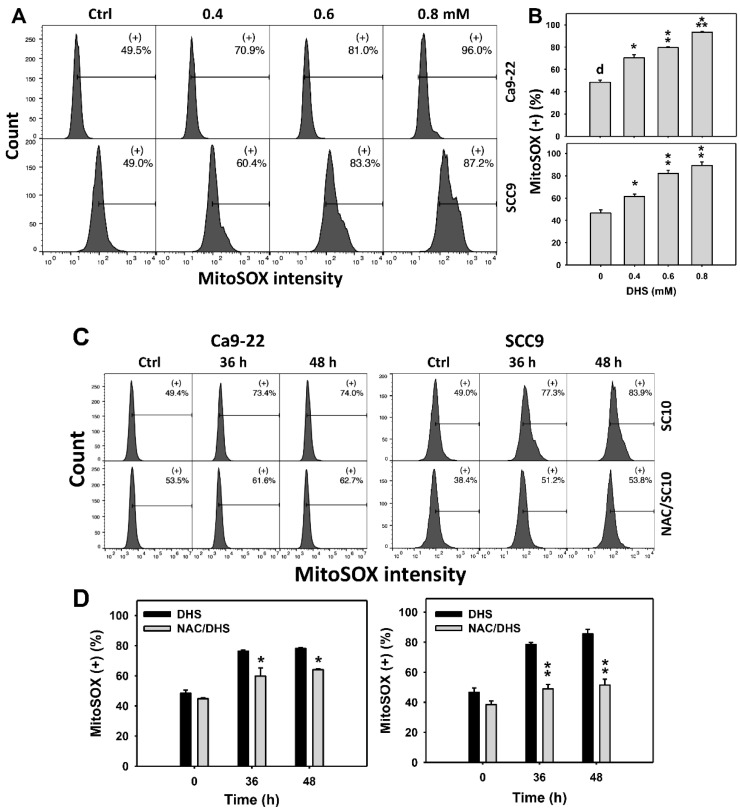
DHS effects on MitoSOX contents of oral cancer cells. (**A**,**B**) Representative MitoSOX patterns of DHS-treated oral cancer cells. Cells (Ca9-22 and SCC-9) were treated with 0.4, 0.6 and 0.8 mM DHS or vehicle (containing 0.08% DMSO) for 48 h. (+) indicates the high MitoSOX populations. (**C**,**D**) Alleviation of the MitoSOX changes of DHS-treated oral cancer cells by NAC. Cells were pretreated with NAC (4 mM, 1 h) or not, and then they were treated with 0.6 mM DHS or vehicle for 0, 36, and 48 h. Data = means ± SDs (*n* = 3 independent experiments). *, ** and *** indicate significant differences between (**A**,**B**) DHS and control and (**C**,**D**) DHS and NAC/DHS (*p* < 0.05, 0.01 and 0.001, respectively). A positive control for MitoSOX generation of oral cancer cells is provided in Supplementary Figure S1.

Since MitoSOX is commonly involved in triggering apoptosis [30], the MitoSOX induction of DHS-treated oral cancer cells was evaluated. The concentration effect of DHS on the MitoSOX pattern in oral cancer cells was demonstrated (Figure 6A). The MitoSOX (+) events of oral cancer cells (Ca9-22 and SCC-9) were increased by DHS treatment (Figure 6B). Moreover, the impact on the time course of MitoSOX changes in oral cancer cells following DHS treatment was demonstrated (Figure 6C). The MitoSOX (+) events of Ca9-22 and SCC-9 cells were increased by DHS treatment from 0 to 48 h (Figure 6D). After NAC pretreatment, the DHS-induced MitoSOX stresses were slightly suppressed in Ca9-22 cells but dramatically suppressed in SCC-9 cells, suggesting that DHS induced MitoSOX stress in oral cancer cells (SCC-9) in a ROS-dependent manner.

### 2.7. DHS Induces MitoMP Depletion Stress of Oral Cancer Cells Alleviated by NAC

**Figure 7:**
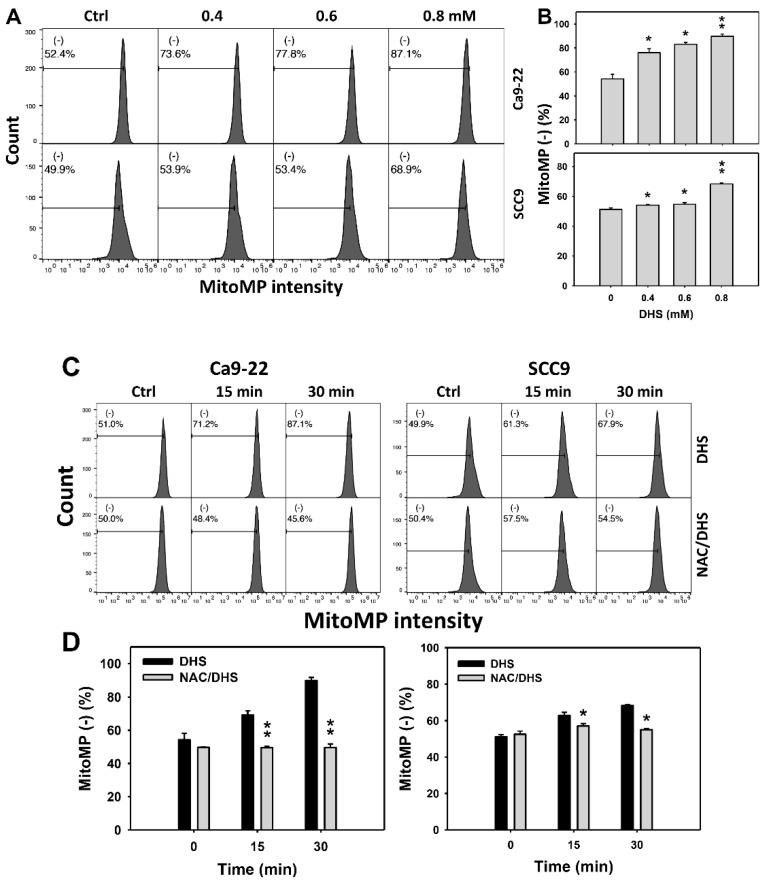
DHS effects on MitoMP contents of oral cancer cells. (**A**,**B**) Representative MitoMP patterns of DHS-treated oral cancer cells. Cells (Ca9-22 and SCC-9) were treated with 0.4, 0.6 and 0.8 mM DHS or vehicle (containing 0.08% DMSO) for 48 h. (−) indicates the low MitoMP populations. (**C**,**D**) Alleviation of the MitoMP changes of DHS-treated oral cancer cells by NAC. Cells were pretreated with NAC (10 mM, 1 h) or not, and then they were treated with 0.6 mM DHS or vehicle for 0, 36 and 48 h. Data = means ± SDs (*n* = 3 independent experiments). * and ** indicate significant differences between (**A**,**B**) DHS and control and (**C**,**D**) DHS and NAC/DHS (*p* < 0.05 and 0.01, respectively). A positive control for MitoMP depletion of oral cancer cells is provided in Supplementary Figure S1.

Since MitoMP is commonly involved in triggering apoptosis [31], the MitoMP depletion of DHS-treated oral cancer cells was evaluated. The concentration effect of DHS on the MitoMP pattern in oral cancer cells was demonstrated (Figure 7A). The MitoMP (−) events of oral cancer cells (Ca9-22 and SCC-9) were increased by DHS treatment (Figure 7B). Moreover, the NAC impact on the time course of MitoMP changes in oral cancer cells following DHS treatment were demonstrated (Figure 7C). The MitoMP (−) events of Ca9-22 and SCC-9 cells were increased from 0 h to 48 h by DHS treatment (Figure 7D). After NAC pretreatment, the DHS-induced MitoMP depletion stress was dramatically suppressed in Ca9-22 cells but only slightly suppressed in SCC-9 cells, suggesting that DHS induced MitoMP depletion stress in oral cancer cells (Ca9-22) in a ROS-dependent manner.

### 2.8. DHS Induces γH2AX Phosphorylation of Oral Cancer Cells Alleviated by NAC

**Figure 8:**
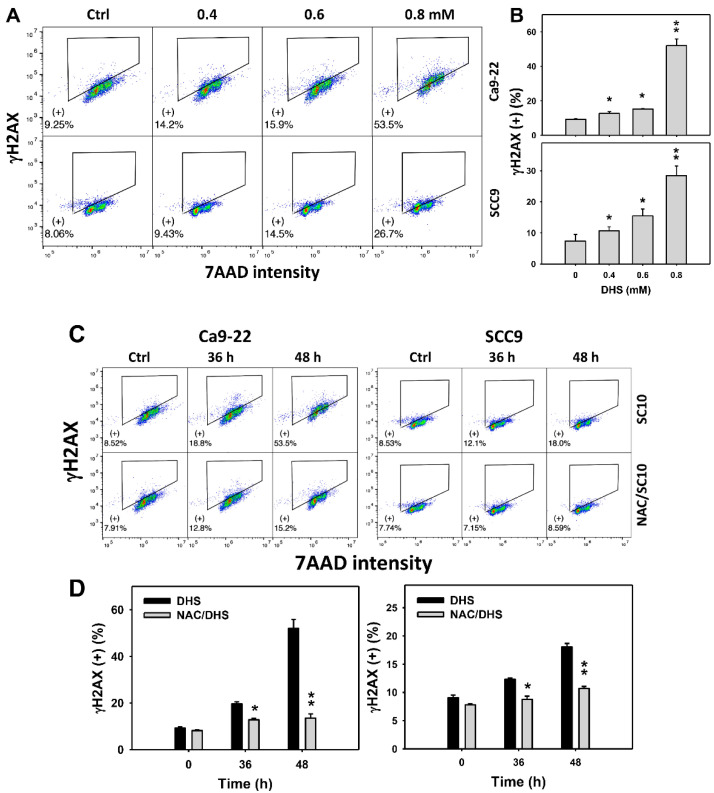
DHS effects on γH2AX contents of oral cancer cells. (**A**,**B**) Representative γH2AX patterns of DHS-treated oral cancer cells. Cells (Ca9-22 and SCC-9) were treated with 0.4, 0.6, and 0.8 mM DHS or vehicle (containing 0.08% DMSO) for 48 h. (+) indicates the high γH2AX populations. (**C**,**D**) Alleviation of the γH2AX changes of DHS-treated oral cancer cells by NAC. Cells were pretreated with NAC (10 mM, 1 h) or not, and then they were treated with 0.6 mM DHS or vehicle for 0, 36 and 48 h. Data = means ± SDs (*n* = 3 independent experiments). * and ** indicate significant differences between (**A**,**B**) DHS and control and (**C**,**D**) DHS and NAC/DHS (*p* < 0.05 and 0.01, respectively). A positive control for γH2AX phosphorylation of oral cancer cells is provided in Supplementary Figure S1.

Since DNA damage may cause MitoSOX generation and apoptosis [32], γH2AX-detected DNA damage of DHS-treated oral cancer cells was evaluated. The concentration-dependent effect of DHS on the γH2AX pattern in oral cancer cells was demonstrated (Figure 8A). The γH2AX (+) events of oral cancer cells (Ca9-22 and SCC-9) were increased by DHS treatment (Figure 8B). Moreover, the NAC impact on the time course of γH2AX changes in DHS-treated oral cancer cells following DHS treatment were demonstrated (Figure 8C). The γH2AX (+) events of Ca9-22 and SCC-9 cells were increased from 0 to 48 h by DHS treatment (Figure 8D). After NAC pretreatment, the DHS-induced γH2AX phosphorylations were suppressed, suggesting that DHS induced γH2AX phosphorylation in oral cancer cells in a ROS-dependent manner.

## 3. Discussion

The pharmaceutical effects of DHS were rarely investigated. Recently, DHS demonstrated an anti-inflammatory effect, which was validated by suppressing the inducible nitric oxide synthase (iNOS) expression in lipopolysaccharide-stimulated RAW264.7 macrophages [33]. However, the anticancer effects of DHS remain unclear. Most DHS studies provide only the purification and cytotoxicity information without addressing detailed antiproliferation mechanisms. The present study confirmed the antiproliferation effect of DHS on oral cancer cells, and its potential mechanisms were examined.

### 3.1. Oxidative Stress Effect of DHS

Cell survival is maintained by balancing between cellular oxidative stress and the antioxidant machinery. During short-term oxidative stress, antioxidants may tolerate this stress and keep redox homeostasis. However, drug-induced oxidative stress may have long-term effects and possibly a sustained perturbation of redox homeostasis [34].

DHS exhibits antioxidant activity [26]. In general, antioxidants have dual functions for cell proliferation. Antioxidants may prevent oxidative stress at low concentrations but induce oxidative stress and cell killing at high concentrations [35]. Accordingly, DHS may exhibit a cell-killing effect on oral cancer cells. In the present study, oxidative stress was analyzed by flow cytometry. DHS induces ROS generation, MitoSOX production, and MitoMP depletion in oral cancer cells. Accordingly, DHS may be considered a ROS modulating agent. This warrants a more detailed investigation of antioxidant responses to DHS-induced oxidative stress in the future.

### 3.2. Antiproliferation Effect of DHS against Oral Cancer Cells

Antioxidants can result in a bifunctional modification of oxidative stress [35]. Antioxidants at physiological concentrations may alleviate cellular ROS. In contrast, antioxidants at cytotoxic concentrations may elevate cellular ROS and exhibit ROS-modulating effects.

Some ROS-modulating agents were developed to exhibit antiproliferation effects for anticancer therapy and show little cytotoxicity to normal cells [36,37]. Since DHS has antioxidant abilities [26], DHS is a potential ROS-modulating agent. The principle is based on exogenous ROS that may exceed the ROS threshold of cancer cells with high levels of endogenous ROS. At the same time, exogenous ROS is tolerated by normal cells, providing low endogenous ROS levels [38]. Normal cells are either not or less affected, which explains the lack of/low side effects of DHS treatment. Literature related to DHS is rare [20,27,39]. In 1977, the IC_50_ value of DHS for lymphocytic leukemia P-388 cells was reported as 3.27 nM [20]. In 2003, the IC_50_ values of human lung cancer (A549), colon cancer (HT-29), and leukemia (HL-60) cells were reported as greater than 0.15 mM [27]. However, the detection time of the MTT assay and the cytotoxic mechanisms of DHS were not investigated in that study [28].

In the present study, the IC_50_ values of DHS for oral cancer cells (Ca9-22, OECM-1, CAL 27, and SCC-9) in 48 h MTS assay were: 0.39, 0.69, 0.8 and 0.65 mM, respectively (Figure 1A). Although it shows higher IC_50_ values in oral cancer cells than previously tested cancer cells, its cytotoxicity in non-malignant oral cells is concerning. In comparison, the clinical drug cisplatin showed an IC_50_ value of 27 μM to CAL 27 cells [40]. Although DHS is less sensitive to oral cancer cells than cisplatin, cisplatin is frequently associated with severe side effects. Importantly, the non-malignant oral cells (HGF-1) remained in a healthy condition after 48 h of DHS treatment. Therefore, we report here for the first time that DHS has antiproliferative potential in oral cancer cells without having cytotoxic effects on non-malignant oral cells. It also warrants detailed screening for the DHS antiproliferative responses to other cancer cell models in the future.

Except for an extra conjugated double bond in sinularin, both sinularin and DHS have similar structures [26]. This conjugated double bond may contribute to the higher antioxidant and cytotoxic functions of sinularin compared to DHS. In 24 h ATP assays, the IC_50_ values of sinularin were 32, 2 and 12 μM for breast, lung, and liver cancer cells (MDA-MB-231, H1299, and HA22T/VGH), respectively; whereas the IC_50_ values of DHS were 60, 70 and 120 μM, respectively [26]. Although DHS showed lower cytotoxicity than sinularin, DHS still exhibited anti-oral cancer effects without cytotoxic effects on non-malignant oral cells.

In addition, non-malignant control (HGF-1) and oral cancer cells (OC-2 and HSC-3) increased cell viability after treatment with DHS at concentrations of 0.1–0.4 and 0.1–0.2 mM, respectively. At high concentrations, DHS decreased the cell viability of oral cancer cells. This phenomenon may partly be attributed to the hormesis effect [41] whereby low exposure shows stimulation and high exposure shows inhibition effects of drugs. However, the non-malignant control did not show an inhibition response to DHS at a high concentration (0.8 mM). The other oral cell lines do not display this hormesis effect by lacking the low concentration stimulation response but showing a high concentration inhibition response regarding cell viability.

### 3.3. DHS Induced Apoptosis and Causes DNA Damage Effects on Oral Cancer Cells

Oxidative stress can modulate and trigger apoptosis [34,42]. In the present study, DHS induces oxidative stress and leads to apoptosis by causing a subG1 accumulation (Figure 2), increasing annexin V intensity (Figure 3), and activating caspase signaling (Figure 4C–E). Moreover, signals of annexin and 7AAD can indicate early or late apoptosis or necrosis by annexin V (+)/7AAD (−), annexin V (+)/7AAD (+) or annexin V (−)/7AAD (+), respectively [43]. In the present study, late apoptosis is dominant compared to early apoptosis and necrosis in DHS-treated oral cancer cells. In addition, caspase 3 (+) intensities were increased in oral cancer cells following DHS treatment. Therefore, caspase 3 in apoptosis signaling was activated by DHS. Moreover, both intrinsic and extrinsic apoptosis signaling proteins are known to regulate drug-induced apoptosis. It warrants detailed investigation for exploring the role of intrinsic and extrinsic apoptosis signaling with proteins such as caspases 9 and 8 in DHS-treated oral cancer cells.

Oxidative stress also triggers several kinds of DNA damage [44], such as double-strand breaks and oxidative DNA damage. γH2AX is a marker for DNA double-strand breaks [45,46]. In response to DHS-induced oxidative stress, the flow cytometry-detected γH2AX phosphorylation was induced by DHS in oral cancer cells.

Moreover, oxidative stress is known to cause protein damage and suppress DNA repair [47,48]. For example, ROS inhibits base excision repair proteins, such as 8-oxoguanine-DNA glycosylase (OGG1) [49]. This warrants a detailed examination of the DNA repair modulating potential of DHS in the future.

### 3.4. Oxidative Stress Is Important in DHS-Induced Antiproliferation Mechanisms on Oral Cancer Cells

As shown in this study, DHS provided the following effects: induced antiproliferation, apoptosis (subG1 accumulation, annexin V detection, and pancaspase activation), ROS generation, MitoSOX production, MitoMP depletion, and DNA damage (γH2AX). A NAC pretreatment suppressed all these effects, but it may show differential regulations between two oral cancer cell lines (Ca9-22 and SCC-9). For example, NAC dramatically suppressed antiproliferation, subG1 accumulation, annexin V-detected apoptosis, and γH2AX-detected DNA damage in Ca9-22 and SCC-9 cells. However, NAC dramatically suppressed ROS and MitoSOX generation in SCC-9 cells but suppressed MitoMP depletion in Ca9-22 cells. For pancaspase detection, NAC consistently suppressed DHS-induced pancaspase activation in both cell lines only at 48 h treatment. Accordingly, some of the DHS-induced oxidative stress variables may be cell line-specific. Asking for the mechanism of action behind the observed effects, our results suggest that the above DHS-induced effects were mediated by oxidative stress. However, the mechanisms for differential regulations of oxidative stress variables in different oral cancer cell lines remain unclear and warrant detailed investigation.

### 3.5. The Influence of Toxicity on Apoptosis, Oxidative Stress, and DNA Damage in Oral Cancer Cells

As described above, Ca9-22 cells were 1.67-fold more sensitive to DHS than SCC-9 cells based on IC_50_ values. After 48 h of treatment with 6 mM DHS, Ca9-22 cells showed higher pancaspase and caspase 3 activations, MitoMP depletion, and γH2AX phosphorylation than SCC-9 cells. Other detections such as sub-G1 accumulation, annexin V, ROS, and MitoSOX showed similar effects to DHS treatments for these oral cancer cells. These differential regulations to DHS may depend on cell type characteristics, i.e., Ca9-22 and SCC-9 cells being derived from gingival and tongue tumor tissues, respectively.

Recently, the strategy of combined anticancer treatments has been proposed. In general, low concentrations of potential drugs were combined to sensitize the drug response to clinical drugs. For example, resveratrol [50] and berberine [51] have been combined with cisplatin for synergistic antiproliferation to lung and ovarian cancer cells. In the present study, DHS showed high IC_50_ values at 48 h MTS assay, suggesting a high concentration of DHS was required for anti-oral cancer application, which may raise the potential in vivo toxicity problem. This must be considered, especially for further potential clinical use. The high concentration of DHS may also limit its application as a combined treatment with clinical drugs. Alternatively, extending the exposure time to lower concentrations of DHS may favor the cytotoxicity rates in cancer cells while reducing the potential toxicity of DHS. Therefore, it warrants detailed evaluations of combining a low concentration of DHS with other anticancer drugs for anti-oral cancer therapy.

## 4. Materials and Methods

### 4.1. Preparation of DHS and Inhibitors

By following the procedure described in our previous study [18], the crude product yielded from the ethyl acetate extraction of the lyophilized bodies of the soft coral *S. manaarensis* was chromatographed to produce DHS at a high purity, as proven by ^1^H and ^13^C NMR spectra (Supplementary Figures S2 and S3). The inhibitor, NAC (Sigma-Aldrich, St. Louis, MO, USA) [28,52,53,54], was pretreated before DHS treatment.

### 4.2. Cell Culture and Viability

Five oral cancer cell lines (Ca9-22, SCC-9, CAL 27, OC-2, and HSC-3) and one non-malignant oral cell line (HGF-1) were purchased from ATCC (Manassas, VA, USA) and Health Science Research Resources Bank (HSRRB) (Osaka, Japan). Oral cancer cell line OECM-1 was kindly provided by Dr. Wan-Chi Tsai (Kaohsiung Medical University, Kaohsiung, Taiwan) [55]. These cell lines were derived from the gingival (Ca9-22, OCEM-1, and HGF-1), tongue (SCC-9, CAL 27, and HSC-3), and buccal (OC-2) locations. The cultural conditions were as described in [56]. CellTiter 96 Aqueous One Solution, a mitochondrial enzyme reacting kit, was applied to determine cell viability (Promega, Madison, WI, USA) [55,57].

### 4.3. Cell Cycle

After 75% ethanol fixation, cells were incubated with 7AAD (1 μg/mL, 30 min) (Biotium, Inc., Hayward, CA, USA) [58,59]. The cellular DNA content was detected by Accuri C6 flow cytometer (Becton-Dickinson, Mansfield, MA, USA), and cell cycle phases were identified by Flow Jo software (Ashland, OR, USA). The sub-G1 phase, one of the apoptosis phenomena, was calculated.

### 4.4. Apoptosis Assays by Annexin V/7AAD, Pancaspase, and Caspase 3

Annexin V can detect phosphatidylserine in the leaflet of the plasma membrane of apoptotic cells. Annexin V/7AAD kit (Strong Biotech Corporation, Taipei, Taiwan) was used to measure apoptosis [56,60]. FITC-labeled annexin V (10 μg/mL) and 7-AAD (1 μg/mL) were mixed for cell incubation for 30 min at 37 °C. Pancaspase activity kit (Abcam; Cambridge, UK) was used to measure the activities for Cas-1 and Cas-3 to 9. Cells were incubated with 0.2X Tide Fluor^TM^ 2-Val-Ala-Asp-fluoromethyl ketone for 2 h. For caspase 3 activation assay, activated caspase 3 was reacted with 10 μM substrate solution (1:1000) at 37 °C for 1 h using OncoImmunin kits (Gaithersburg, MD, USA) [61]. Annexin V/7AAD, pancaspase, and caspase 3 intensities were measured using an Accuri C6 flow cytometer. Detailed procedures were performed according to the user manual.

### 4.5. Oxidative Stress Assays for ROS, MitoSOX, and MitoMP

Oxidative stress induces changes in ROS, MitoSOX, and MitoMP, which were detected by several specific chemical probes. For the ROS assays, cells were incubated with 2’,7’-dichlorodihydrofluorescein diacetate (DCFH-DA; 2 μM, 30 min) (Sigma-Aldrich; St. Louis, MO, USA) [56]. The intracellular ROS level was proportional to DCFH-DA intensity, being detected by the Accuri C6 flow cytometer. For the MitoSOX assay, cells were incubated with MitoSOX™ Red (5 μM, 30 min) (Molecular Probes, Invitrogen, Eugene, OR, USA) [56]. The mitochondrial superoxide level was proportional to MitoSOX Red intensity, which was detected by the Accuri C6 flow cytometer. For the MitoMP assay, cells were incubated with DiOC_2_(3) (50 nM, 20 min) in MitoProbe^TM^ kit (Invitrogen, San Diego, CA, USA) [56]. The MitoMP level was proportional to DiOC_2_(3) intensity.

### 4.6. DNA Damage Assays by γH2AX Phosphorylation

Since γH2AX is a protein for targeting DNA double-strand breaks, monitoring the relationship of γH2AX and cellular DNA is reasonable. For the γH2AX assay, p-Histone H2A.X antibody (Santa Cruz Biotechnology, Santa Cruz, CA, USA) was used to recognize the phosphorylated H2AX (γH2AX). Then, the Alexa Fluor^®^488-secondary antibody (Cell Signaling Technology, Danvers, MA, USA) was applied [62]. 7AAD (1 μg/mL, 30 min) was further applied to stain cellular DNA. Both γH2AX and 7AAD (+) populations were examined for DNA damage with double-strand breaks. The Accuri C6 flow cytometer detected the Alexa Fluor^®^488 intensities for γH2AX contents.

### 4.7. Statistical Analysis

One-way analysis of variance (ANOVA) with Tukey’s HSD post hoc test [63] was applied to evaluate significances (JMP12 of SAS Institute; Cary, NC, USA). *, ** and *** indicate significant differences between drug treatments and control (*p* < 0.05, 0.01 and 0.001, respectively). Each detection was based on three independent experiments.

## 5. Conclusions

The anticancer effects of DHS have seldom been investigated to date. In the present study, we firstly validated that DHS exhibits an antiproliferation effect on oral cancer cells without cytotoxicity towards non-malignant oral cells. DHS induces several types of cellular oxidative stresses such as ROS affecting MitoSOX generation and MitoMP depletion. DHS also triggers apoptosis, double-strand breaks, and oxidative DNA damages. Mechanistically, all these antiproliferation effects and responses were dependent on oxidative stress. Its antiproliferative effects make DHS a potential anti-oral cancer drug without cytotoxic effects on non-malignant oral cells.

## Data Availability

Data are contained within the article.

## Supplementary Materials

The following are available online at https://www.mdpi.com/article/10.3390/ph14100994/s1, Figure S1: Positive controls (H_2_O_2_-treated oral cancer cells) for the flow cytometry in Figure 2, Figure 3, Figure 4, Figure 6, Figure 7 and Figure 8. Figure S2: The ^1^H-NMR spectrum of DHS, Figure S3: The ^13^C-NMR spectrum of DHS.
