# Supplementary material for: Soft Coral-Derived Dihydrosinularin Exhibits Antiproliferative Effects Associated with Apoptosis and DNA Damage in Oral Cancer Cells
Source: Pharmaceuticals (Basel). 2021 Sep 29;14(10):994. doi: 10.3390/ph14100994 (PMC8539362; doi:10.3390/ph14100994)
Supplement: Supplementary file 1 [file pharmaceuticals-14-00994-s001.zip › Supplementary Figure- Fig S1- A to L-Positive control.pdf]

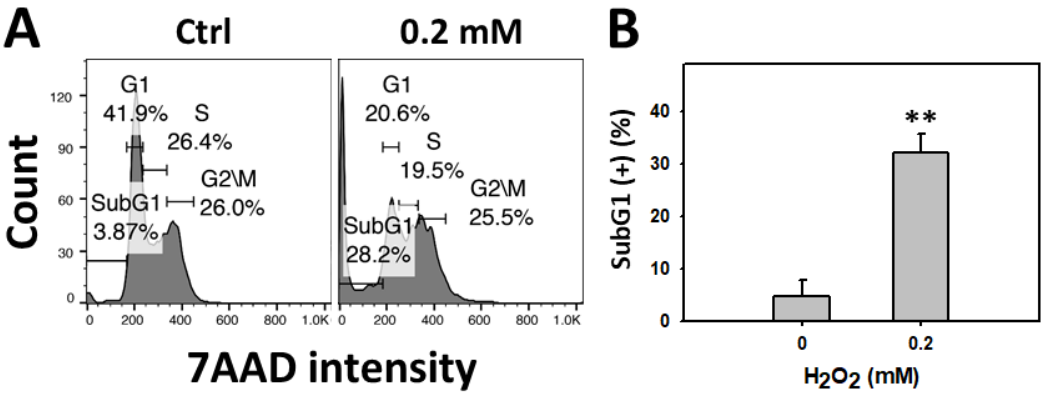

Figure S1-AB. Positive control patterns for subG1 accumulation. (A) Flow cytometry pattern. (B) Statistics. Cells (Ca9-22) were treated with H_2O_2 (200 μM) for 24 h. ** indicates $P < 0.01$ (t-test). Data = mean ± SD (n = 3).

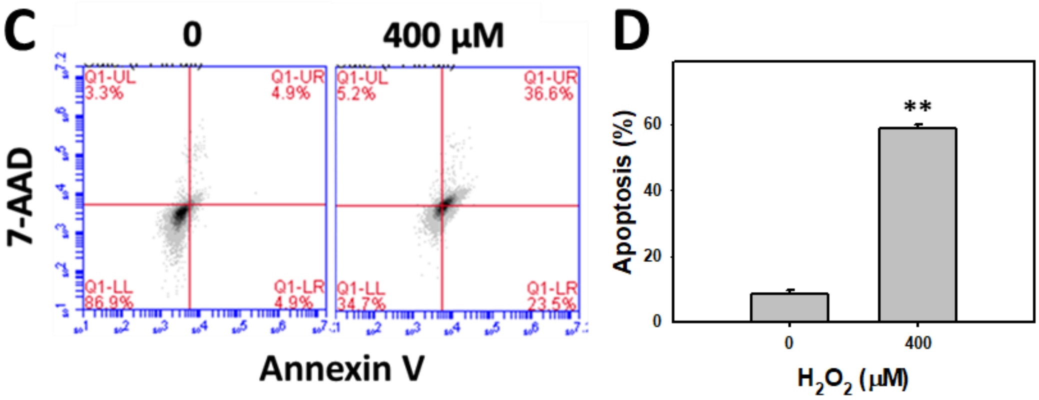

Figure S1-CD. Positive control patterns for apoptosis. (C) Flow cytometry pattern. (D) Statistics. Cells (Ca9-22) were treated with H_2O_2 (400 μM) for 24 h. ** indicates $P <$ 0.01 (t-test). Data = mean ± SD (n = 3).

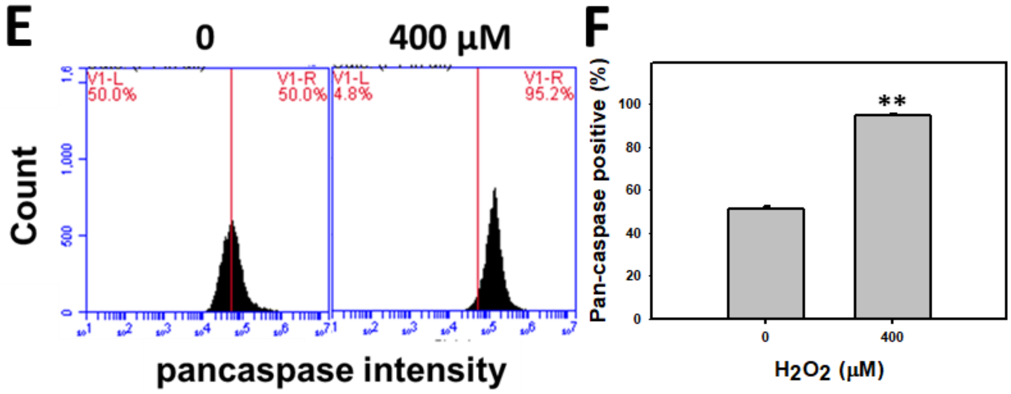

Figure S1-EF. Positive control patterns for pancaspase. (E) Flow cytometry pattern. (F) Statistics. Cells (CAL 27) were treated with H_2O_2 (400 μM) for 24 h. * and ** indicate $P < 0.05$ and $P < 0.01$ (t-test). Data = mean ± SD (n = 3).

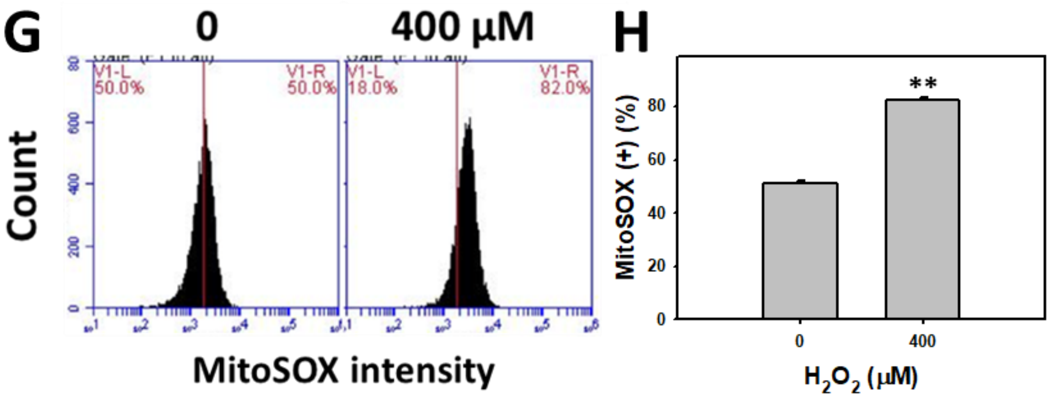

Figure S1-GH. Positive control patterns for MitoSOX analysis. (G) Flow cytometry pattern. (H) Statistics. Cells (Ca9-22) were treated with H_2O_2 (400 μM) for 24 h. ** indicates $P < 0.01$ (t-test). Data = mean ± SD (n = 3).

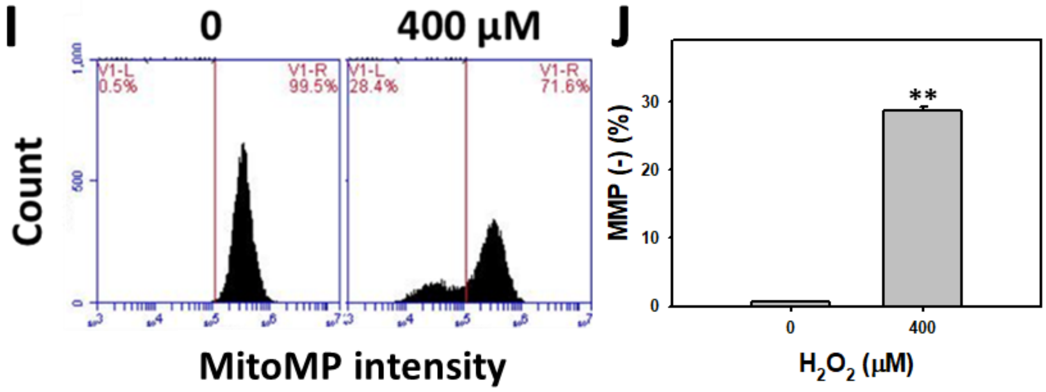

Figure S1-IJ. Positive control patterns for MitoMP analysis. (I) Flow cytometry pattern. (J) Statistics. Cells (Ca9-22) were treated with H_2O_2 (400 μM) for 24 h. ** indicates $P < 0.01$ (t-test). Data = mean ± SD (n = 3).

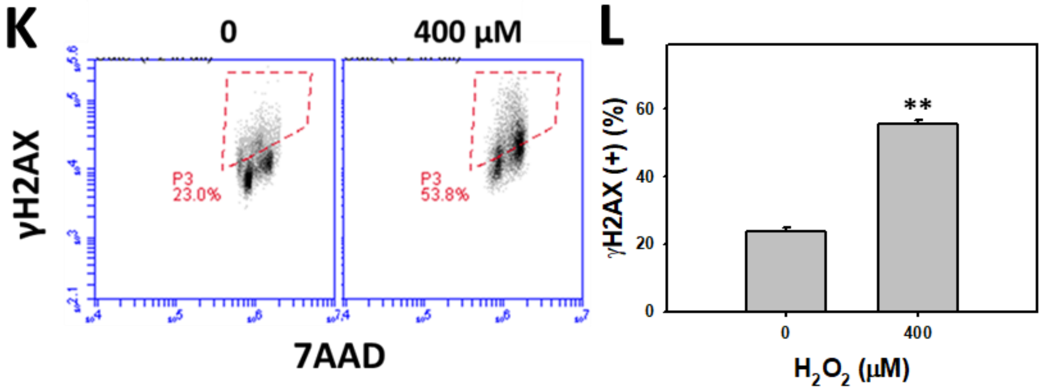

Figure S1-KL. Positive control patterns for γH2AX analysis. (K) Flow cytometry pattern. (L) Statistics. Cells (Ca9-22) were treated with H_2O_2 (400 μM) for 24 h. ** indicates $P < 0.01$ (t-test). Data = mean ± SD (n = 3).
