# Supplementary figures and images for: Soft Coral-Derived Dihydrosinularin Exhibits Antiproliferative Effects Associated with Apoptosis and DNA Damage in Oral Cancer Cells
Source: Pharmaceuticals (Basel). 2021 Sep 29;14(10):994. doi: 10.3390/ph14100994 (PMC8539362; doi:10.3390/ph14100994)

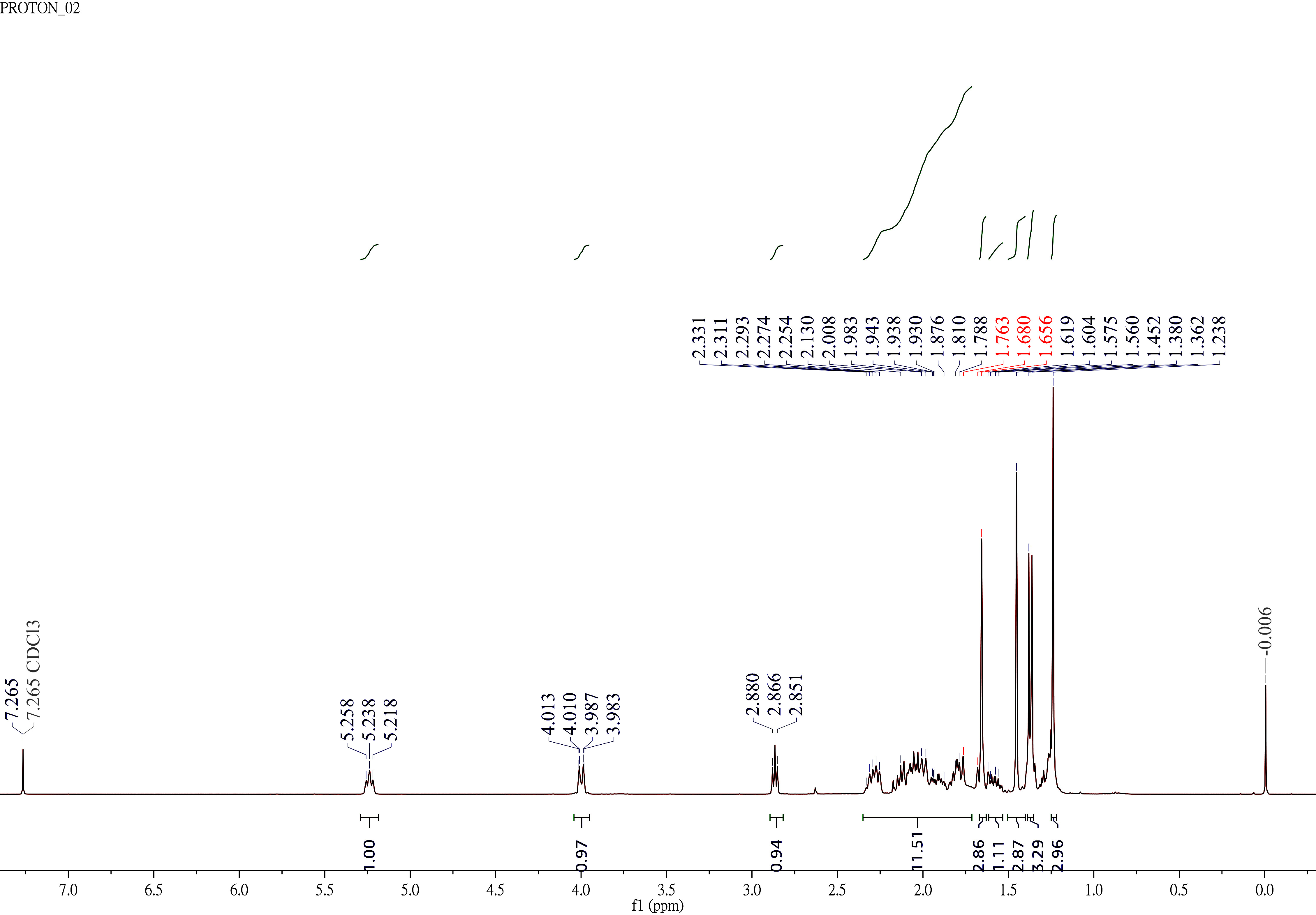

Supplement: Supplementary file 1 [file pharmaceuticals-14-00994-s001.zip › Supplementary Figure S2-Dihydrosinularin-proton.tif]

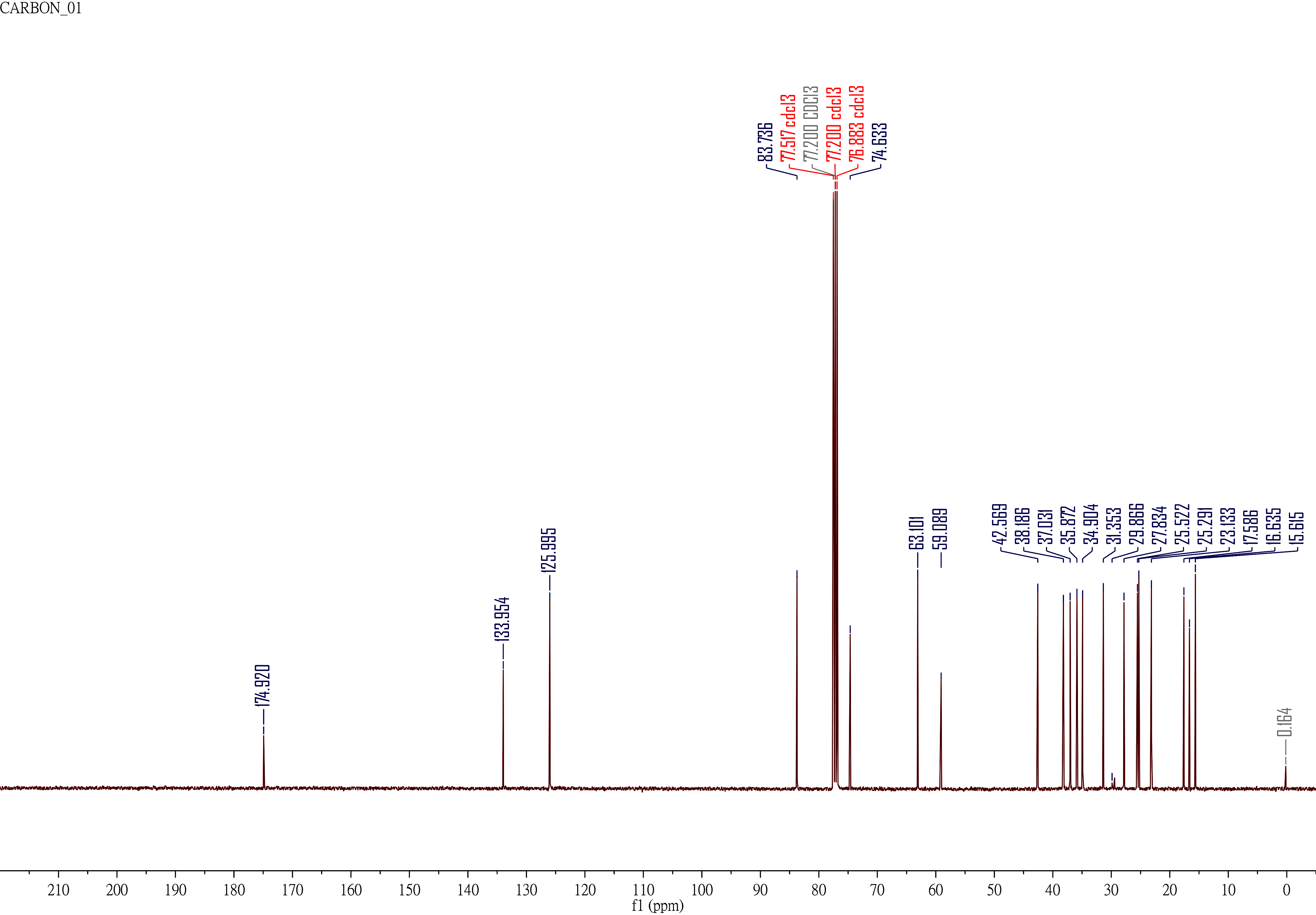

Supplement: Supplementary file 1 [file pharmaceuticals-14-00994-s001.zip › Supplementary Figure S3-Dihydrosinularin-carbon.tif]
